# The Begomovirus Disease Tetrahedron: Weeds as the Missing Dimension in Virus Epidemiology

**DOI:** 10.3390/v18060647

**Published:** 2026-06-04

**Authors:** Marjia Tabassum, Thuy T. B. Vo, Nattanong Bupi, Muhammad Amir Qureshi, Hyo-Jin Im, Min-Kwan Kim, Imankul Assem, S. M. Hemayet Jahan, Li-Long Pan, Giuseppe Parrella, Peter Palukaitis, Taek-Kyun Lee, Sukchan Lee

**Affiliations:** 1 Department of Integrative Biotechnology, Sungkyunkwan University, Suwon 16419, Republic of Korea; marjia39@g.skku.edu (M.T.); bichthuy251188@gmail.com (T.T.B.V.); gum.bupi@gmail.com (N.B.); amirq303@gmail.com (M.A.Q.); imhyoj99@gmail.com (H.-J.I.); yjdhhome2@skku.edu (M.-K.K.); asem.iman03@gmail.com (I.A.); 2 Department of Entomology, Patuakhali Science and Technology University, Dumki, Patuakhali 8602, Bangladesh; hemayet_pstu@yahoo.com; 3 Ministry of Agriculture Key Laboratory of Molecular Biology of Crop Pathogens and Insect Pests, Zhejiang Key Laboratory of Biology and Ecological Regulation of Crop Pathogens and Insects, Institute of Insect Sciences, Zhejiang University, Hangzhou 310058, China; panlilong@zju.edu.cn; 4 Institute for Sustainable Plant Protection of the National Research Council (IPSP-CNR), 80055 Portici, Italy; giuseppe.parrella@ipsp.cnr.it; 5 Jeonbuk National University, Jeonju 56443, Republic of Korea; scripath1@yahoo.co.uk; 6 Ecological Risk Research Department, Korea Institute of Ocean Science & Technology, Geoje 53201, Republic of Korea

**Keywords:** begomoviral epidemiology, weed reservoirs, virus–vector–host interactions, disease tetrahedron framework

## Abstract

Begomoviruses are among the most destructive plant viruses, causing substantial yield losses across diverse cropping systems. Their epidemiological success is driven by high genetic plasticity, broad host range, and efficient transmission by the whitefly vector *Bemisia tabaci*. Traditional epidemiological models based on the classical disease triangle (virus–host–vector) fail to fully capture the ecological and evolutionary complexity of begomovirus pathosystems. Increasing evidence highlights the critical role of non-cultivated plants, particularly weeds, as persistent reservoirs that maintain viral populations during off seasons, facilitate recombination, and act as primary inoculum sources for subsequent outbreaks. Here, we propose the Begomovirus Disease Tetrahedron, an integrative framework that expands the disease triangle by incorporating weeds as a fourth essential component. We synthesize current knowledge on begomovirus adaptive evolution, including genome plasticity, noncanonical protein functions, and virus–vector mutualism, alongside key ecological drivers such as seasonal dynamics, agricultural intensification, and landscape connectivity. By integrating molecular, ecological, and epidemiological perspectives, this framework provides a comprehensive understanding of begomovirus emergence and persistence, offering new insights for the development of sustainable and ecologically informed disease management strategies.

## 1. Introduction

Plant virus epidemics are increasingly recognized as complex, multi-component systems shaped by dynamic interactions among viruses, hosts, vectors, and the surrounding agroecosystem. Among these, begomoviruses (family *Geminiviridae*) represent one of the most economically devastating groups of plant viruses, causing severe yield losses in a wide range of crops, particularly in tropical and subtropical regions [1,2]. Their remarkable evolutionary success is largely due to high mutation rates, frequent recombination, and their intimate association with the whitefly vector *Bemisia tabaci*, which facilitates rapid dissemination across diverse plant hosts [3].

Traditionally, plant virus epidemiology has been conceptualized using linear or triangular models that emphasize interactions between the virus, host, and vector. However, accumulating evidence indicates that such frameworks inadequately capture the ecological and evolutionary complexity of begomovirus pathosystems. In particular, non-cultivated plants, especially weeds, play a pivotal yet underappreciated role as persistent reservoirs, enabling virus survival during off-seasons and acting as sources of primary inoculum for subsequent crop infections [4,5]. These reservoirs not only sustain viral populations but also create opportunities for recombination, selection, and the emergence of novel variants with enhanced virulence or host adaptability.

Recent studies further demonstrate that the interaction between begomoviruses and *B. tabaci* is not merely vector-mediated transmission but involves a finely tuned mutualistic relationship that enhances vector fitness and virus spread [6]. Concurrently, agricultural intensification, climate variability, and landscape heterogeneity have amplified the connectivity between cultivated and non-cultivated hosts, thereby accelerating the epidemiological cycles of begomoviruses.

Despite these advances, there remains a lack of a unifying conceptual framework that integrates these interconnected components into a coherent model. To address this gap, we propose the Begomovirus Disease Tetrahedron, an integrative framework that expands the classical disease triangle by incorporating weeds as a fourth critical dimension. This model conceptualizes begomovirus epidemics as a dynamic system driven by reciprocal interactions among the virus, host plants, whitefly vectors, and reservoir weeds across spatial and temporal scales. By synthesizing molecular, ecological, and epidemiological insights, this framework provides a more comprehensive understanding of virus persistence, emergence, and spread.

In this review, we examine the ecological and evolutionary foundations of begomovirus–host–vector interactions, with particular emphasis on the role of weeds as hidden reservoirs and drivers of disease dynamics. We further explore how these interactions shape viral diversity, transmission pathways, and epidemic outbreaks, ultimately highlighting the implications for sustainable disease management and future research directions.

## 2. The Adaptive Landscape of Begomoviruses

**Figure 1:**
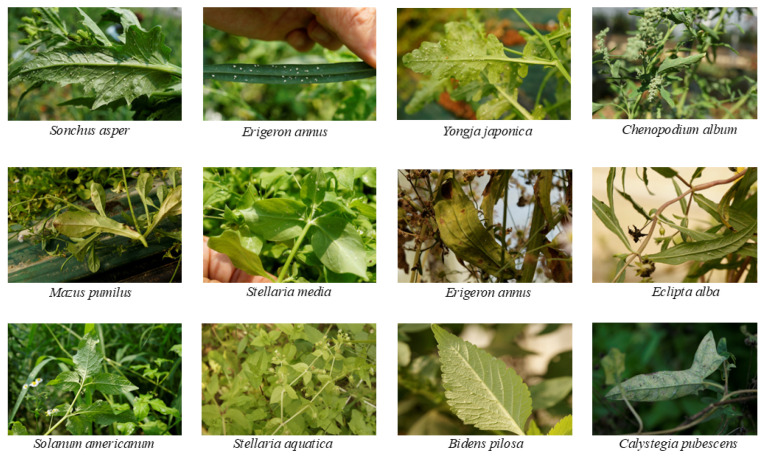
Representative weeds that serve as the host of Whitefly and TYLCV. Images were captured using a 55 mm zoom lens under natural light. All weeds were photographed at similar distances to maintain visual consistency. Relative leaf and insect sizes can be inferred accordingly.

All living entities exist within a complex web of independence, relying on one another for survival and dispersion. The success of begomoviruses stems from their remarkable ability to adapt to and develop across diverse environments. This adaptability is driven by frequent genome recombination, which continuously generates new variants and species, and by the help of DNA satellites, some of which act as pathogenicity determinants, further enhancing their virulence [7,8,9,10,11]. Begomoviruses infect a broad range of cultivated and wild hosts, allowing them to spread efficiently and persist in various ecosystems. Their insect vectors exhibit a broad spectrum of host plants, facilitating the transmission of these viruses across different plant species. Nonetheless, several environmental factors facilitate and affect various stages of the smooth infection cycle of this destructive virus group. Figure 1 shows representative weeds acting as reservoir hosts for *Bemisia tabaci* and tomato yellow leaf curl virus (TYLCV). A study conducted using naturally growing weeds for TYLCV, tomato chlorosis virus (ToCV), and whitefly infestation. Among 37 weed species comprising 93 individual plants from 16 families tested, 21 supported whitefly proliferation, 16 tested positive for ToCV, and 24 were infected with TYLCV—with 11 species harboring both viruses and whiteflies were reported. To validate these findings, a greenhouse experiment was conducted using 59 weed species from 22 families infested with TYLCV-positive whiteflies. The results revealed that 38 species supported whitefly proliferation, 14 were TYLCV-positive, and 11 served as hosts for both viruses and whiteflies [12].

A growing body of research has shown that begomovirus genomes encode a number of unusual, noncanonical proteins whose functions are becoming clearer, in addition to the canonical group of five to eight proteins. For example, the monopartite ageratum leaf curl Sichuan virus’ C5 protein increases virulence, viral replication, and silencing suppression [13], while the bipartite mungbean yellow mosaic India virus’ (MYMIV) AC5 protein functions as a virulence determinant and suppressor of RNA silencing [14]. In a recent study, the translation initiation sites (TISs) within the genome of tomato yellow leaf curl Thailand virus were mapped experimentally [15]. Specifically, two downstream TISs within the AV2 ORF led to the production of different protein forms with distinct subcellular localization patterns. Intriguingly, mutations in these AV2 isoforms resulted in significantly reduced disease symptoms, highlighting their importance in the viral infection process. Moreover, recent studies also have revealed hidden loci in the virus genomes that can encode important proteins, suggesting that there is still much to learn about how these viruses encode proteins. Collectively, these findings emphasize that begomovirus genomes harbor more genetic information than previously understood. Hidden ORFs and multifunctional proteins expand the virus’ toolkit for adaptation, reinforcing their evolutionary success and necessitating deeper genomic analyses to uncover additional elements involved in pathogenesis.

## 3. Epidemiological Framework of Emerging Plant Viruses

Over the past two decades, there has been a notable increase in the incidence of infectious diseases affecting humans, domestic and wild animals, as well as plants, because of the emergence of new pathogens and the resurgence of previously identified ones. This trending phenomenon has sparked significant interest in emerging pathogens, defined as “the causative agents of infectious diseases whose incidence is increasing following their appearance in new host populations or whose incidence is increasing in existing host populations due to long-term changes in the underlying epidemiology” [16,17].

The foundation of the cause of plant virus emergence is a very complex matter. Up to nine different scenarios was considered that favor plant virus emergence, which can be summarized into four groups: (i) changes in the host plant and/or virus ecology; (ii) changes in the genetic composition of the host populations; (iii) changes in the genetic composition of the virus population; and (iv) in the case of vectored viruses, changes in the ecology and/or genetic composition of the vector [4].

The time when environmental conditions are ideal for plant growth is referred to as the growing season or on season. Perennials endure for several seasons, biennials for two seasons, and annuals for one season. Most crops need at least 90 frost-free days during the growing season, which is usually determined by either the number of days with temperatures above the species-specific growth threshold or the number of frost-free days between the last spring frost and the first autumn or winter frost. The off-season refers to the time interval between the post-harvest season and the start of a new planting. Normally, at this time, weather conditions are not so favorable for the main crops. Therefore, some rural producers take the opportunity to make improvements on the farm and prepare the soil for the next harvest. The off-season occurs right after the harvest, and the term was adopted because the conditions of climate, temperature, luminosity, and humidity are a little lower than those that occur during the harvest (https://husqvarna-water.com/off-season-farming-three-key-benefits-and-the-crucial-role-of-irrigation/ accessed on 26 March 2026).

Generally, during emergence, viruses originate from their well-established host species that play the role of reservoir hosts. During spreading from the reservoir into a new environment and causing productive infections, many steps are required. Factors affecting the emergence of viruses consist of three phases: In Phase I, the virus must transit from the reservoir population to infect individuals of either the same host species in a new ecological environment, or a new host species. In Phase II, the virus must adapt to the new host or environment, so that infections are productive enough to allow sustained transmission between individuals of the (new) host. In Phase III, the epidemiology of the virus must change to optimize transmission in the (new) host population, which often requires adaptation to new vector species or new modes of transmission [18].

During the cropping season, natural insect vectors, economically important crops, and undesired plants (weeds), all go through favorable environments to grow. Many studies report the occurrence of *B. tabaci* on crops as well as on weeds near fields or greenhouse areas. Population outbreaks and challenging field management are extremely common issues posed by whiteflies, owing to their high reproduction rate and short development period [19].

The majority of the *B. tabaci* species complex members may transmit most, if not all, begomoviruses, according to several studies [20,21]. Nevertheless, the transmission efficiency of the various *B. tabaci* taxonomic range greatly [22,23,24,25,26]. Even within populations of the same species, variations in transmission efficiency have been noted [27]. For instance, the transmission effectiveness of chino del tomate virus (CdTV) or TYLCV by MEAM1, MED, and other *B. tabaci* species varies up to tenfold [28,29,30], although the transmission efficiency of TYLCSV is constant across species [25]. distinct life history features [31], eating habits, host plant preferences [20,25,32,33], bacterial endosymbionts carried by whiteflies [30,34,35], and the genetic background of the insect population may all contribute to the varying transmission capacities of distinct *B. tabaci* species. The genetic basis of insects most likely controls.

At least two bacterial species—*Hamiltonella defensa*, which is found in specialized insect cells called bacteriomes [30,35], and *Rickettsia* spp., which occupies the insect’s entire body cavity have been linked to the transmission of TYLCV [34]. Symbiotic bacteria have been shown to have significant effects on *B. tabaci* [36]. A 63 kDa GroEL protein (formerly known as symbionin) that interacts with TYLCV virions during route to the salivary glands has been demonstrated to be encoded by *Hamiltonella*. This interaction is hypothesized to protect virions from the insect immune system, thereby increasing the likelihood that the virus will be efficiently transmitted [30,35].

By comparing the transmission efficiency of Rickettsia-infected and uninfected *B. tabaci* genetic sister strains, a recent study [34] has linked *Rickettsia* to *B. tabaci*-TYLCV interactions. In comparison to an uninfected strain, this study showed that the Rickettsia-infected strain received more TYLCV from infected plants, maintained the virus longer, and showed nearly double the transmission efficiency. Interestingly, there was an inverse correlation between TYLCV and Rickettsial levels in the whiteflies, indicating an antagonistic link between the virus and the bacteria within the insect [34].

**Figure 2:**
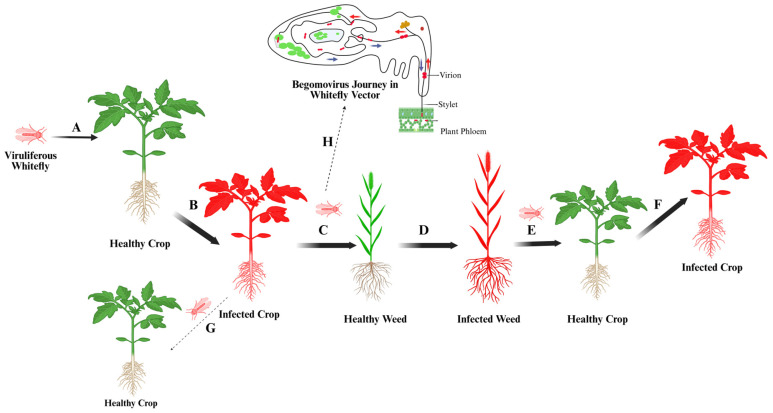
**Seasonal dynamics of weed hosts in begomovirus–whitefly–crop interactions.** (**A**) Viruliferous *Bemisia tabaci* transmit begomoviruses to healthy crop plants. (**B**) Infected crops serve as primary virus sources during the growing season. (**C**) Whiteflies acquire the virus from infected crops and subsequently feed on nearby healthy weeds. (**D**) Weeds become secondary reservoirs following virus acquisition. (**E**) Whiteflies transmit the virus from infected weeds back to crops. (**F**) Newly infected crops amplify the virus–vector cycle. (**G**) Virus spreads horizontally among crop plants through continuous whitefly-mediated transmission. (**H**) Schematic representation of begomovirus movement within the whitefly body, from ingestion to subsequent inoculation into plant phloem.

As depicted in Figure 2, *Bemisia tabaci* mediates the continuous exchange of begomoviruses between crop and weed hosts, ensuring viral maintenance during the cropping season.

## 4. The Mutualistic Relationship Between Begomoviruses and *B. tabaci*

For the last twenty years, research has shown that whiteflies, comprising over 1500 species belonging to 161 genera worldwide, are among the primary vectors responsible for transmitting plant viruses among plants [37]. Whiteflies use a series of ingestion, circulation, and release processes to spread persistent viruses. The stylet absorbs virions during feeding, which then travel through the midgut into the hemolymph and build up in the primary salivary glands before being injected into fresh plants via saliva (Figure 2). After entering the plant, the virus multiplies and spreads throughout the entire system, resulting in symptoms like stunting, yield loss, and yellowing. Viral evolution may be aided by mutations or recombination brought on by interactions between viral particles and whitefly receptor proteins during transmission. Also, it is mentioned that latent period before begomovirus transmission is roughly eight hours, and symptoms appear two to four weeks after infection [38].

The capsid protein (CP) of begomoviruses is the key translocator in *B. tabaci* [38]. CP is located at the surface of the virion and remains in contact with various insect tissues starting from ingestion to egestion. CP from different sources, such as non-transmissible begomoviruses and in vitro mutated CP, helped to identify amino acids that are critical for virus transmission. Overall, the CP ensures virus translocation inside the whitefly.

The CP also plays a crucial role in distinguishing between viruses that can cross the gut/hemolymph barrier and those that cannot [39]. Altering specific residues within the CP region, spanning amino acids 129 to 152, restored the infectivity of naturally non-transmissible isolates of tomato yellow leaf curl Sardinia virus (TYLCSV) [40]. In addition, the exchange of three amino acids in the CP resulted in efficient whitefly-mediated transmission of a non-transmissible abutilon mosaic virus isolate [41].

The relationship between begomoviruses and whiteflies can be described as mutualistic, as demonstrated in various studies. It was demonstrated that feeding on tomato yellow leaf curl China virus (TYLCCNV)-infected tobacco plants significantly altered the transcriptional profiles of female adult *Bemisia tabaci*, as revealed by next-generation sequencing and quantitative real-time PCR analyses [42]. Genes involved in the oxidative phosphorylation pathway and detoxification enzymes were downregulated in MEAM1 individuals feeding on virus-infected plants. This reduced detoxification activity probably lowered energy costs and boosted whitefly performance. These results pointed to the mechanisms of plant-mediated whitefly-virus mutualism.

## 5. Weeds as Begomovirus Reservoirs

Although typically regarded as agricultural nuisances, weeds can harbor and perpetuate begomoviruses between cropping cycles, providing continuous sources of infection that threaten tomato and other economically important crops. In the 1940s, yellow leaf curl-like symptoms were observed for the first time in tomato plants due to an outbreak of sweet potato whitefly populations, and later, it was shown that the presence of a begomovirus, TYLCV, was the cause of the infection [43]. Subsequently, TYLCV infections were reported on weeds, such as *Euphorbia* sp., *Lamium amplexicaule*, *Malva parviflora*, and *Ageratum conyzoides*, and that weeds growing in Southeast Asia were found infected with both monopartite/satellite complexes and bipartite begomoviruses [44,45]. During the cropping season, non-viruliferous whiteflies suck the cell sap from virus-infected plants, become the carriers of the virus and transmit the virus to other plants near the cultivating area. In a Korean study, 28 weed samples collected from the area around greenhouses of tomato farms were infected with TYLCV [46]. They also confirmed the weed infection by constructing an infectious TYLCV clone and later checking the weeds by PCR. Eventually, during the TYLCV spread, weeds become the intermediate host to make the next generation of whiteflies viruliferous. *Solanum nigrum* served as a recombination vessel to produce viral recombinants, induced with curling on leaf margins, leaf chlorosis, growth stunting, and whole-leaf crumpling and the complete nucleotide sequence confirmed the infection by tomato leaf curl Palampur virus [47]. Overall, these studies shed light on how insect vectors transit begomovirus from the main crop to their alternative host and how the latter eventually become the reservoir and inoculum source of the next virus outbreak in economic crop production (Figure 2).

Designing and implementing ecological weed management strategies at the level of the agroecosystem is challenging. It requires a detailed understanding of complex ecological interactions and theoretically relevant practices to meet the needs and constraints of the range of environments and farming systems around the world, while weeds are associated with the most dangerous plant virus group known [48]. The propagation of susceptible cultivars to diversify cropping systems and the seasonal dominance of whitefly vectors have been demonstrated as the major contributors to the occurrence of yellow mosaic disease [49]. Moreover, the weeds of legume fields were reported to serve as alternative hosts and virus reservoirs during the off-season. The weed, *A. conyzoides*, was shown to harbor MYMIV and TLCV [49,50]. Thus, increasing evidence suggests that weed plants may serve as permanent sources of infection and may have a role in disease epidemiology (Figure 2).

**Figure 3:**
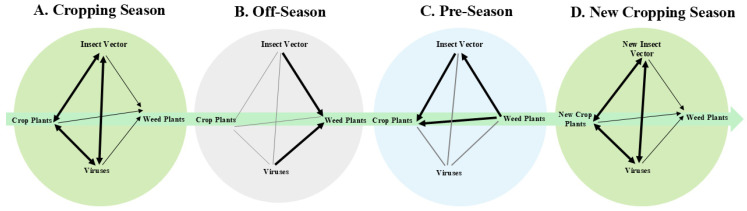
**The begomovirus disease tetrahedron.** (**A**) **Cropping-season transmission:** During the cropping season, begomoviruses are disseminated from infected crop plants to neighboring non-crop hosts through the whitefly vector (*Bemisia tabaci*). At this stage, crops constitute the principal host reservoir, while weeds and other non-crop plants may become incidentally infected through vector-mediated transmission. The simultaneous presence of susceptible crops, vectors, viruses, and alternative hosts facilitates local virus spread within the agroecosystem. (**B**) **Off-season persistence:** Following crop harvest or during periods when cultivated hosts are absent, weeds and other non-crop plants function as alternative reservoirs for both begomoviruses and their whitefly vectors. By maintaining viral inoculum and vector populations during unfavorable periods, these hosts play a critical role in preventing local extinction and ensuring pathogen survival between cropping cycles. (**C**) **Pre-Season Initiation of new epidemics:** With the onset of a new cropping season and the return of favorable environmental conditions, viruliferous whiteflies originating from infected weed reservoirs colonize newly planted crops and introduce the virus into susceptible seedlings. This primary inoculum source initiates new infection within the crop population. (**D**) **Epidemiological continuity across seasons:** The recurrent movement of begomoviruses between cultivated crops and weed reservoirs, mediated by whitefly vectors, sustains a continuous virus–vector–host network across successive growing seasons. This cyclical process promotes pathogen persistence, facilitates disease emergence, and contributes to the maintenance and recurrence of begomovirus epidemics at both local and regional scales.

The survival of inoculum between two cropping seasons mainly occurs in the seeds or other organs of alternative hosts, which grow actively near the main crop [51]. In this regard, it is essential to highlight that when preferred hosts are not present during off-season periods, weeds assist in the maintenance and survival of the pest populations in the cultivation area, which eventually contributes to the continuity of the virus and insect cycle [52]. Seasonal transitions of begomovirus epidemics, encompassing cropping season transmission, off-season persistence, and reinitiation of infection, are summarized in the Begomovirus Disease Tetrahedron (Figure 3).

What makes these weeds so stubborn in nature? There are many growth characteristics and adaptations that allow them to win over crops. Synchronized germination, rapid establishment and growth of seedlings, tolerance to adverse weather conditions, immediate response to available soil moisture and nutrients, tolerance to shading created by the crop plants, and resistance to herbicides are some of the most crucial traits possessed by weeds that allow them to occupy a favorable ecological niche.

Both weeds and crops belonging to the same families have many botanical similarities such as susceptibility to many begomoviruses. Weeds from the *Cucurbitaceae* family are known to be the wild ancestors of the familiar plants that are cultivated for foods nowadays. *Momordica charantia* is a wild cucurbit weedy species that can act as a reservoir of squash vein yellowing virus, a whitefly-transmitted virus that causes watermelon vine decline, a serious disease of squash and watermelon [53]. In addition to that, about 24 species of begomoviruses and their variations worldwide are reported to be able to infect cucurbits that includes both crop and non-crops. The most common ones are the tomato leaf curl Palampur virus, squash leaf curl China virus, and tomato leaf curl New Delhi virus (ToLCNDV) [54].

Several cultivated and non-cultivated solanaceous species have been identified as natural hosts of Tomato leaf curl New Delhi virus (ToLCNDV), including *Solanum lycopersicum*, *S. melongena*, *S. tuberosum*, and *S. nigrum*. The ability of ToLCNDV to infect both crop and weed hosts highlights the potential role of solanaceous weeds as virus reservoirs, facilitating viral survival during cropping gaps and contributing to disease epidemiology through vector-mediated transmission [55,56,57,58].

An important question arises as to why are these families very much prone to begomovirus infection? The factor of being from the same family as the crop plants can be one of the reasons; however, further studies at the molecular level are required for confirmation.

By changing microbial, insect, and viral communities and serving as ecological links between cultivated and arid areas, weeds have a substantial impact on cropland ecosystems. Weeds increase microbial activity and draw pollinators and insects through symbiotic relationships, habitat creation, and nutrient cycling. Significant nutrient uptake by weeds has been documented in several studies; *Parthenium hysterophorus*, *Abutilon theophrasti*, and others have demonstrated high absorption of nitrogen, phosphorus and potassium [59,60,61]. Weeds badly affect the growth of their surrounding crops, owing to the allelochemicals released from them [62]. Allelopathic interactions are mostly species-specific and can go both ways [63]. Weeds such as nutsedges, crabgrass, Canada thistle, and spotted knapweed are known to release allelochemicals toxic to many crops. On the other hand, winter rye, mustards (*Brassica* spp.), forage radish (*Raphanus sativus*), and sorghum–Sudan grass (*Sorghum bicolor* × *sudanense*), which are cultivated crops, can defeat many weeds through allelopathy [63,64,65].

**Figure 4:**
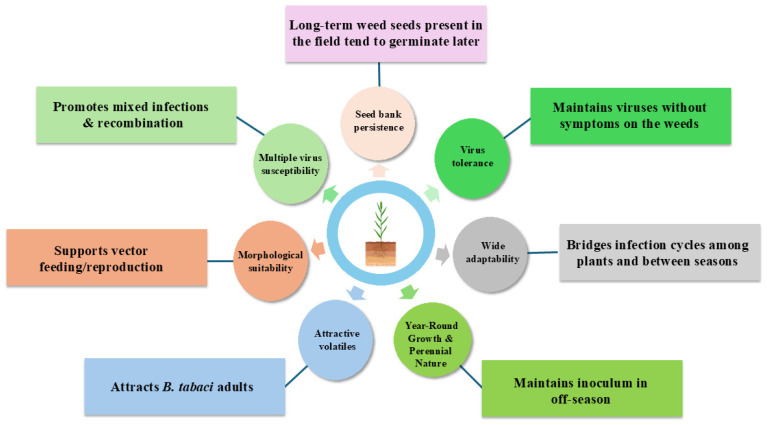
Key ecological traits of weed hosts facilitating their role as reservoirs for begomoviruses and whitefly vectors.

As illustrated in Figure 4, several ecological traits enable weeds to function as effective reservoirs for begomoviruses and their whitefly vectors. Many weedy species exhibit year-round growth or a perennial habit, allowing both viruses and vectors to persist in the absence of cultivated hosts. Their high susceptibility to diverse begomoviruses facilitates mixed infections and viral exchange within the weed community. Morphological features such as soft leaf surfaces and dense canopies provide favorable microhabitats for whitefly colonization and reproduction. Additionally, the emission of specific volatile compounds enhances vector attraction, promoting virus acquisition and transmission. The rapid growth and long-term persistence of weed seed banks further ensure continual regeneration of host populations, while their tolerance to virus infection enables sustained viral maintenance without severe symptom expression [3,66,67,68,69,70,71].

## 6. Weeds as the Origin of Pathogenic Transmission: Investigation into the Source of Viral Infection

Knowledge regarding the source of primary inoculum is essential for developing effective disease management strategies in the event of a disease epidemic. When the primary source of inoculum is known, the risk of losses by the pest infestation can be mitigated. Among the most important facts of the plant host–begomovirus–insect vector relationship is that the main hosts, known to be cultivated crops, are only present in the fields for a certain period, and in their absence, alternative plants serve as the reservoirs of the virus and the vector for a few months. The virus is then retransmitted by its vector, the whitefly (*B. tabaci*), to the main host crop after it is recultivated. Although the sources of the primary inoculum of the begomoviruses are still not completely annotated, significant works have been performed throughout the years to focus on the diversity and spreading of these dangerous viruses [72]. The whitefly *B. tabaci* transmits begomoviruses and inoculates into plant cells in a circulative persistent manner that includes a very precise virus–host interaction, subsequently resulting in begomovirus infection.

As mentioned earlier, crops are not constantly in the field, so from general logic, viruses should disappear from the field, although they manage to come back every season and cause severe losses in agriculture and the economy. When a susceptible host and aggressive pathogen interact under favorable conditions, a disease occurs and eventually turns into an epidemic. Humans practice agriculture in a monoculture base which allows the continuous presence of plant hosts in the field, specifically mentioned for begomoviruses which prefer not only crops but also weeds as their host [72]. When weeds become a reservoir of both viruses and vectors, viruses spread and infestation in the following season may be extremely severe and devastating [73]. Whiteflies are known to be abundant in tropical and subtropical regions, but recently they have been reported to have a great impact and pose a severe threat to a variety of greenhouse crops in temperate regions. Whiteflies were found abundantly inside and outside of the greenhouses during and after the cropping season, implying that during the off-season outsiders hosting the pest become the next season inoculum source for begomoviral infection [74].

### Weeds as Drivers of Begomovirus Emergence

*Jatropha curcas* is a shrub-like weed of NW origin, with yellow mosaic symptoms, collected from three geographical locations in the Dominican Republic (DO). Sequence comparison of the associated begomovirus showed the highest identities with DNA A component of jatropha mosaic virus (JMV). The study showed jatropha infected with JMV strains in the DO could be the source of genetic diversity for the evolution of crop-infecting begomoviruses and a potential reservoir. Studies revealed that JMV not only infects jatropha, but also common bean and tobacco plants, in which symptoms were produced [75].

Three species of weeds (two *A. conyzoides*, one *Sonchus oleraceus*, and one turnip), from Pakistan and Nepal, showing typical begomovirus symptoms, were analyzed for begomovirus identification by sequence analysis of full-length begomovirus clones and betasatellite clones [76]. The sequence analysis revealed ageratum enation virus (AEV) isolates with greater than 89.1% nucleotide sequence identity. Infectivity analyses performed using clones of two AEV isolates, along with the beta satellites, to agroinoculate *N. benthamiana*, *N. tabacum*, *Solanum lycopersicum*, and *A. conyzoides*, showed that AEV is a virus from weeds that has a predominant capacity to infect crops. A new begomovirus named Corchorus mottle virus (CoMoV), was found in Orinoco jute (*Corchorus hirtus*), and characterized by sequence analysis and phylogenetic data [77]. CoMoV showed the closest relationship with abutilon mosaic Brazil virus, with an identity of 87.3% for DNA-A. After inoculation, using a biolistic method and an infectious clone of CoMoV, *S. rhombifolia* produced typical symptoms, whereas *N. benthamiana* and tomato plants remained symptomless, despite being infected.

*P. hystrophorus* and *Sonchus asper*, two very common invasive species of weeds in India, were found naturally infected by TLCV in Uttar Pradesh, but the plants were symptomless. The whitefly *B. tabaci* transmitted the virus from these infected weeds to tomato plants. After 15–25 days of inoculation, tomato plants were expressing symptoms of leaf curling and twisting [78].

*Sida* plants showing yellow mosaic symptoms were infected by sida yellow mosaic Gujarat virus, along with a betasatellite (ludwigia leaf distortion betasatellite) and an alpha satellite (malvastrum yellow mosaic alpha satellite) in Gujarat, India, demonstrating that weeds harbored a begomovirus–alphasatellite–betasatellite complex [79]. The authors stated that this weed host acts as a potential source of virus inoculum and whiteflies can transmit viruses from weeds to other cultivated crops.

Research has shown very elaborate and extensive evidence that weeds are the source of inoculum for further infection of crop plants [45]. They reported *L. amplexicaule* as a source of TYLCV and also demonstrated the full scenario where whiteflies could transmit viruses from infected weeds to healthy tomato plants. Further research performed with *Eleusine indica* demonstrated that weeds can act as an inoculum source of TYLCV for whitefly-mediated transmission [46]. In both cases, TYLCV-infected tomato plants showed typical symptoms. Virus detection from tomato plants, weeds, and insects was done via both PCR using specific primers and Southern blot hybridization for more specificity.

## 7. Conclusions

Begomovirus epidemics are increasingly understood as complex, multi-component systems shaped by continuous interactions among viruses, host plants, insect vectors, and the surrounding agroecosystem. In this review, we propose the Begomovirus Disease Tetrahedron as a unifying conceptual framework that expands the classical disease triangle by incorporating weeds as a fourth critical dimension. This model captures the spatiotemporal continuity of begomovirus pathosystems, emphasizing that disease persistence is sustained not only within crops but also across non-cultivated plant communities.

Weeds emerge as pivotal drivers of begomovirus epidemiology, functioning as long-term reservoirs that maintain viral inoculum and support populations of the whitefly vector *Bemisia tabaci* during off-season periods. Their ecological traits, including perennial growth, asymptomatic infection, and capacity to harbor mixed viral populations, facilitate recombination, diversification, and the emergence of novel variants with enhanced adaptive potential. Concurrently, the mutualistic interactions between begomoviruses and *B. tabaci* further amplify transmission efficiency and epidemic spread across heterogeneous landscapes.

The tetrahedral framework highlights that begomovirus outbreaks are the result of interconnected ecological and evolutionary processes operating across seasonal and landscape scales. Importantly, it underscores the need to move beyond crop centric management approaches toward integrated strategies that explicitly incorporate weed ecology and reservoir dynamics.

Future research should prioritize mechanistic understanding of virus weed compatibility, quantitative assessment of reservoir contributions to epidemic initiation, and the influence of environmental change on virus–vector–host interactions. Collectively, integrating these dimensions will be essential for developing predictive models and sustainable management strategies to mitigate the global impact of begomovirus diseases.

## Data Availability

No new data were created or analyzed in this study. Data sharing is not applicable to this article.
